# Bacterial biosynthetic efficiency is constrained by cell geometry and intracellular diffusion

**DOI:** 10.64898/2026.07.25.740728

**Published:** 2026-07-27

**Authors:** Arianna Cylke, Indrajit Badvaram, Shiladitya Banerjee

## Abstract

Bacterial metabolic strategies are tied to cell size and shape, yet how geometry constrains the efficiency of biomass production is not well understood. Here we develop a coarse-grained whole-cell model of bacterial physiology that couples proteome allocation, metabolic fluxes, and cell geometry to physical limits on surface area and intracellular diffusion. We define the biosynthetic energy efficiency as the fraction of ATP available from imported carbon that is invested in biomass, and find that it is non-monotonic in nutrient availability, peaking precisely at the onset of overflow metabolism. This identifies the metabolic switch as an optimal trade-off between efficient use of imported carbon and rapid growth. Perturbing cell morphology away from the empirical scaling laws shows that increasing surface area at a fixed volume raises both growth rate and efficiency, so the observed size and shape relations sit close to an efficiency optimum rather than being arbitrary. When the empirical growth laws are relaxed and geometry is treated as a free parameter, the model predicts a hard physical limit: the maximum sustainable cell size falls as the target growth rate rises. This ceiling arises from a conflict within the finite proteome budget between the cost of fast growth and the cost of large size, the latter set by the slowing of intracellular diffusion. A few physical rules thus delimit the metabolic strategies and size range available to bacterial life.

## Full Text Availability

The license terms selected by the author(s) for this preprint version do not permit archiving in PMC. The full text is available from the preprint server.

